# 4,4′-{[1,1′-Methyl­enebis(naphthalene-2,1-di­yl)]bis­(­oxymethyl­ene)}di­benzo­nitrile

**DOI:** 10.1107/S1600536808007629

**Published:** 2008-03-29

**Authors:** Yu-Yuan Zhao

**Affiliations:** a Ordered Matter Science Research Center, College of Chemistry and Chemical Engineering, Southeast University, Nanjing 210096, People’s Republic of China

## Abstract

There are two independent mol­ecules in the asymmetric unit of the title compound, C_37_H_26_N_2_O_2_. The crystal structure is stabilized by a weak intra­molecular hydrogen bond as well as C—H⋯π inter­actions.

## Related literature

For the application of nitrile compounds in industry, see: Urbina *et al.* (2001 ▶); Jin *et al.* (1994 ▶); Brewis *et al.* (2003 ▶). 
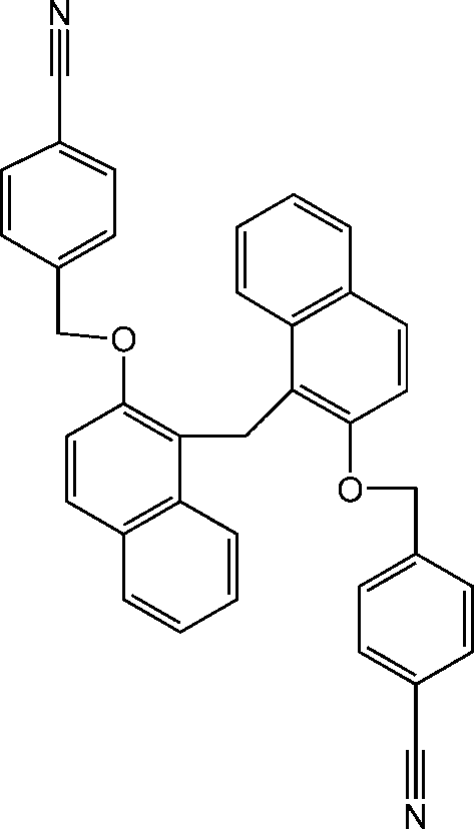


## Experimental

### 

#### Crystal data

C_37_H_26_N_2_O_2_
*M* *_r_* = 530.60
Triclinic, 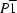

*a* = 14.3054 (10) Å
*b* = 14.4363 (8) Å
*c* = 15.2974 (7) Å
α = 67.7010 (10)°
β = 83.5890 (10)°
γ = 71.7840 (10)°
*V* = 2776.4 (3) Å^3^
*Z* = 4
Mo *K*α radiation
μ = 0.08 mm^−1^
*T* = 293 (2) K
0.20 × 0.20 × 0.12 mm

#### Data collection

Rigaku Mercury2 diffractometer
Absorption correction: multi-scan *CrystalClear* (Rigaku, 2005 ▶) *T*_min_ = 0.799, *T*_max_ = 1.000 (expected range = 0.791–0.991)
25739 measured reflections
10903 independent reflections
5107 reflections with *I* > 2σ(*I*)
*R*_int_ = 0.076

#### Refinement

*R*[*F* ^2^ > 2σ(*F* ^2^)] = 0.070
*wR*(*F* ^2^) = 0.206
*S* = 0.92
10903 reflections
740 parameters
H-atom parameters constrained
Δρ_max_ = 0.20 e Å^−3^
Δρ_min_ = −0.20 e Å^−3^

### 

Data collection: *CrystalClear* (Rigaku, 2005 ▶); cell refinement: *CrystalClear*; data reduction: *CrystalClear*; program(s) used to solve structure: *SHELXTL/PC* (Sheldrick, 2008 ▶); program(s) used to refine structure: *SHELXTL/PC*; molecular graphics: *SHELXTL/PC*; software used to prepare material for publication: *SHELXTL/PC*.

## Supplementary Material

Crystal structure: contains datablocks I, global. DOI: 10.1107/S1600536808007629/bx2133sup1.cif

Structure factors: contains datablocks I. DOI: 10.1107/S1600536808007629/bx2133Isup2.hkl

Additional supplementary materials:  crystallographic information; 3D view; checkCIF report

## Figures and Tables

**Table 1:** Hydrogen-bond geometry (Å, °)

*D*—H⋯*A*	*D*—H	H⋯*A*	*D*⋯*A*	*D*—H⋯*A*
O73—H73*A*⋯O4	0.93	2.44	2.763 (5)	100
C22—H22*A*⋯*Cg*1^i^	0.93	2.88	3.755 (3)	158
C36—H36*A*⋯*Cg*2	0.93	2.57	3.454 (3)	159
C73—H73*A*⋯*Cg*1	0.93	2.78	3.698 (4)	168

Symmetry code: (i) 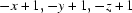. *Cg*1 is the centroid of the C39–C43,C48 ring. *Cg*2 is the centroid of the C2,C3,C8–C11 ring.

**Fig. 1.**
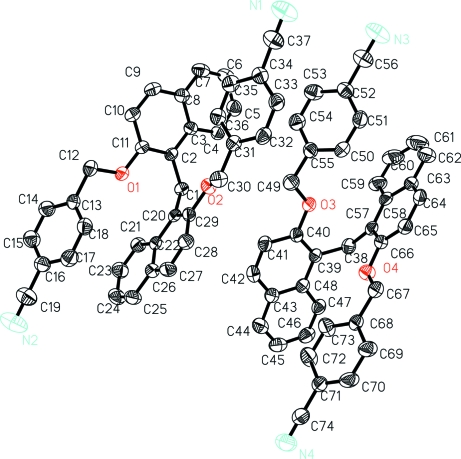
A view of the compound with the atomic numbering scheme. Displacement ellipsoids were drawn at the 30% probability level and all hydrogen atoms were omitted for clarity.

## supplementary crystallographic information

### Comment

The synthesis of new azoles has been a very active area of research and one important aspect has been the incorporation of functional units, such as the cyanomethyl group in ravuconazol (Urbina *et al.*, 2001). Nitrile derivatives have found many industrial applications. For example, phthalonitriles have been used as starting materials for phthalocyanines (Jin *et al.*, 1994), which are important components for dyes, pigments, gas sensors, optical limiters and liquid crystals, and which are also used in medicine, as singlet oxygen photosensitisers for photodynamic therapy (PDT; Brewis *et al.*, 2003). Recently, we have reported a few benzonitrile compounds (Fu & Zhao, 2007). As an extension of our work on the structural characterization, the title compound, is reported here.

The crystal data show that in the title compound, the two naphthyl rings are almost perpendicular to each other and the dihedral angle is 83.25 (7)°. However, the two cyanobenzyloxy rings are approximately parallel with a dihedral angle of 11.27 (1)°. The crystal structure of the title compound, is stabilized by a weak intramolecular hydrogen bond [H73A···.O4 2.44, C73···O4 2.763 (5) Å, O4—H73A···C73 100] as well as C—H -π interactions (Table 1 and Table 2).

### Experimental

1,1'-methylenedinaphthalen-2-ol (0.3 g,1 mmol) and 4-(bromomethyl)benzonitrile (0.392 g,2 mmol) were dissolved in acetone in the presence of K_2_CO_3_ (0.138 g,1 mmol) and refluxed for 3 days. After the mixture was cooled to room temperature, the solution was filtered and rotated in vacuum affording a white precipitate of compound. Colourless crystals of the title compound suitable for X-ray diffraction were obtained from a solution of 100 mg in 15 ml diethylether after 3 days.

### Refinement

All the C—H hydrogen atoms were calculated geometrically and with C—H distances ranging from 0.93 to 0.97Å and were allowed to ride on the C and O atoms to which they are bonded. With which *U*_iso_(H) = 1.2Ueq(C).

### Crystal data

**Table:**

C_37_H_26_N_2_O_2_	*Z* = 4
*M**_r_* = 530.60	*F*_000_ = 1112
Triclinic, *P*1	*D*_x_ = 1.269 Mg m^−^^3^
Hall symbol: -P 1	Mo *K*α radiation λ = 0.71073 Å
*a* = 14.3054 (10) Å	Cell parameters from 19582 reflections
*b* = 14.4363 (8) Å	θ = 3.0–27.5º
*c* = 15.2974 (7) Å	µ = 0.08 mm^−^^1^
α = 67.7010 (10)º	*T* = 293 (2) K
β = 83.5890 (10)º	Block, colorless
γ = 71.7840 (10)º	0.20 × 0.20 × 0.12 mm
*V* = 2776.4 (3) Å^3^	

### Data collection

**Table:**

Rigaku Mercury2 diffractometer	10903 independent reflections
Radiation source: fine-focus sealed tube	5107 reflections with *I* > 2σ(*I*)
Monochromator: graphite	*R*_int_ = 0.076
Detector resolution: 13.6612 pixels mm^-1^	θ_max_ = 26.0º
*T* = 293(2) K	θ_min_ = 3.0º
CCD_Profile_fitting scans	*h* = −17→17
Absorption correction: multi-scanCrystalClear (Rigaku, 2005)	*k* = −17→17
*T*_min_ = 0.799, *T*_max_ = 1.000	*l* = −18→18
25739 measured reflections	

### Refinement

**Table:**

Refinement on *F*^2^	Hydrogen site location: inferred from neighbouring sites
Least-squares matrix: full	H-atom parameters constrained
*R*[*F*^2^ > 2σ(*F*^2^)] = 0.070	*w* = 1/[σ^2^(*F*_o_^2^) + (0.1*P*)^2^] where *P* = (*F*_o_^2^ + 2*F*_c_^2^)/3
*wR*(*F*^2^) = 0.206	(Δ/σ)_max_ = 0.001
*S* = 0.92	Δρ_max_ = 0.20 e Å^−^^3^
10903 reflections	Δρ_min_ = −0.20 e Å^−^^3^
740 parameters	Extinction correction: SHELXL, Fc^*^=kFc[1+0.001xFc^2^λ^3^/sin(2θ)]^-1/4^
Primary atom site location: structure-invariant direct methods	Extinction coefficient: 0.0043 (9)
Secondary atom site location: difference Fourier map	

### Special details

**Table:**

Geometry. All e.s.d.'s (except the e.s.d. in the dihedral angle between two l.s. planes) are estimated using the full covariance matrix. The cell e.s.d.'s are taken into account individually in the estimation of e.s.d.'s in distances, angles and torsion angles; correlations between e.s.d.'s in cell parameters are only used when they are defined by crystal symmetry. An approximate (isotropic) treatment of cell e.s.d.'s is used for estimating e.s.d.'s involving l.s. planes.
Refinement. Refinement of *F*^2^ against ALL reflections. The weighted *R*-factor *wR* and goodness of fit *S* are based on *F*^2^, conventional *R*-factors *R* are based on *F*, with *F* set to zero for negative *F*^2^. The threshold expression of *F*^2^ > σ(*F*^2^) is used only for calculating *R*-factors(gt) *etc*. and is not relevant to the choice of reflections for refinement. *R*-factors based on *F*^2^ are statistically about twice as large as those based on *F*, and *R*- factors based on ALL data will be even larger.

### Fractional atomic coordinates and isotropic or equivalent isotropic displacement parameters (Å^2^)

**Table:**

	*x*	*y*	*z*	*U*_iso_*/*U*_eq_	
O3	0.26966 (14)	0.69880 (16)	0.21213 (13)	0.0634 (6)	
O4	0.36207 (15)	0.81079 (15)	−0.03224 (14)	0.0669 (6)	
C39	0.4200 (2)	0.6345 (2)	0.14721 (19)	0.0509 (7)	
C40	0.3698 (2)	0.6810 (2)	0.2090 (2)	0.0554 (7)	
C43	0.5749 (2)	0.6332 (2)	0.2048 (2)	0.0603 (8)	
C48	0.5251 (2)	0.6063 (2)	0.1465 (2)	0.0560 (8)	
C66	0.2736 (2)	0.7897 (2)	−0.0247 (2)	0.0555 (7)	
C57	0.2725 (2)	0.6903 (2)	0.03295 (19)	0.0538 (7)	
C67	0.3682 (2)	0.9119 (2)	−0.0901 (2)	0.0630 (8)	
H67A	0.3205	0.9640	−0.0695	0.076*	
H67B	0.3527	0.9264	−0.1550	0.076*	
C58	0.1841 (2)	0.6625 (3)	0.0399 (2)	0.0621 (8)	
C65	0.1899 (2)	0.8644 (3)	−0.0758 (2)	0.0669 (9)	
H65A	0.1921	0.9316	−0.1133	0.080*	
C68	0.4692 (2)	0.9192 (2)	−0.0850 (2)	0.0566 (8)	
C41	0.4189 (2)	0.7046 (2)	0.2687 (2)	0.0643 (8)	
H41A	0.3834	0.7351	0.3106	0.077*	
C55	0.1075 (2)	0.7667 (3)	0.2586 (2)	0.0657 (9)	
C71	0.6543 (2)	0.9396 (2)	−0.0771 (2)	0.0626 (8)	
C63	0.1008 (2)	0.7371 (3)	−0.0159 (2)	0.0690 (9)	
C44	0.6786 (2)	0.6109 (3)	0.1998 (2)	0.0746 (10)	
H44A	0.7110	0.6300	0.2366	0.090*	
C38	0.3664 (2)	0.6112 (2)	0.0819 (2)	0.0645 (8)	
H38A	0.4122	0.5982	0.0332	0.077*	
H38B	0.3512	0.5462	0.1181	0.077*	
C64	0.1056 (2)	0.8380 (3)	−0.0703 (2)	0.0730 (10)	
H64A	0.0500	0.8882	−0.1034	0.088*	
C47	0.5846 (2)	0.5528 (2)	0.0903 (2)	0.0679 (9)	
H47A	0.5547	0.5314	0.0535	0.081*	
C42	0.5186 (2)	0.6828 (2)	0.2650 (2)	0.0672 (9)	
H42A	0.5503	0.7011	0.3031	0.081*	
C51	−0.0371 (3)	0.8383 (3)	0.1622 (2)	0.0794 (10)	
H51A	−0.0681	0.8763	0.1035	0.095*	
C50	0.0612 (3)	0.8243 (3)	0.1709 (2)	0.0781 (10)	
H50A	0.0968	0.8532	0.1185	0.094*	
C53	−0.0442 (3)	0.7411 (3)	0.3275 (3)	0.0812 (10)	
H53A	−0.0797	0.7143	0.3809	0.097*	
C49	0.2141 (2)	0.7550 (3)	0.2686 (3)	0.0812 (10)	
H49A	0.2244	0.8235	0.2480	0.097*	
H49B	0.2346	0.7174	0.3342	0.097*	
C52	−0.0907 (2)	0.7957 (3)	0.2409 (3)	0.0694 (9)	
C74	0.7501 (3)	0.9521 (3)	−0.0743 (2)	0.0781 (10)	
C69	0.4996 (3)	0.9965 (3)	−0.1545 (2)	0.0836 (11)	
H69A	0.4581	1.0423	−0.2054	0.100*	
C45	0.7312 (3)	0.5622 (3)	0.1420 (3)	0.0814 (11)	
H45A	0.7991	0.5494	0.1387	0.098*	
C73	0.5313 (3)	0.8544 (3)	−0.0114 (3)	0.0875 (11)	
H73A	0.5111	0.8025	0.0374	0.105*	
C72	0.6233 (3)	0.8633 (3)	−0.0071 (3)	0.0910 (12)	
H72A	0.6647	0.8171	0.0439	0.109*	
C46	0.6847 (3)	0.5314 (3)	0.0881 (2)	0.0823 (11)	
H46A	0.7216	0.4959	0.0502	0.099*	
C54	0.0543 (3)	0.7262 (3)	0.3350 (2)	0.0756 (10)	
H54A	0.0854	0.6876	0.3935	0.091*	
N4	0.8261 (2)	0.9621 (3)	−0.0730 (2)	0.1071 (11)	
C59	0.1737 (3)	0.5637 (3)	0.0984 (3)	0.0874 (11)	
H59A	0.2256	0.5144	0.1378	0.105*	
C56	−0.1935 (3)	0.8123 (3)	0.2335 (3)	0.0918 (12)	
C70	0.5908 (3)	1.0077 (3)	−0.1503 (2)	0.0857 (11)	
H70A	0.6095	1.0620	−0.1974	0.103*	
C62	0.0143 (3)	0.7080 (4)	−0.0128 (3)	0.0978 (13)	
H62A	−0.0401	0.7563	−0.0494	0.117*	
C60	0.0895 (4)	0.5395 (4)	0.0981 (4)	0.1178 (16)	
H60A	0.0853	0.4732	0.1363	0.141*	
N3	−0.2756 (3)	0.8256 (3)	0.2266 (3)	0.1319 (15)	
C61	0.0098 (4)	0.6113 (5)	0.0424 (4)	0.1282 (18)	
H61A	−0.0470	0.5928	0.0429	0.154*	
O2	0.29159 (14)	0.81321 (14)	0.44137 (13)	0.0600 (5)	
O1	0.37325 (14)	0.70793 (16)	0.69447 (14)	0.0655 (6)	
C11	0.2878 (2)	0.6880 (2)	0.68556 (19)	0.0519 (7)	
C20	0.4363 (2)	0.6956 (2)	0.52235 (18)	0.0474 (7)	
C2	0.28969 (19)	0.6397 (2)	0.62340 (18)	0.0469 (7)	
C8	0.1210 (2)	0.6324 (2)	0.6724 (2)	0.0567 (8)	
C21	0.5381 (2)	0.6702 (2)	0.54265 (19)	0.0500 (7)	
C3	0.2070 (2)	0.6061 (2)	0.61961 (19)	0.0503 (7)	
C1	0.38052 (19)	0.6150 (2)	0.56354 (19)	0.0537 (7)	
H1A	0.4262	0.5499	0.6022	0.064*	
H1B	0.3600	0.6027	0.5116	0.064*	
C29	0.3902 (2)	0.7936 (2)	0.45917 (19)	0.0514 (7)	
C31	0.1278 (2)	0.9257 (2)	0.4002 (2)	0.0571 (7)	
C9	0.1215 (2)	0.6889 (2)	0.7301 (2)	0.0636 (8)	
H9A	0.0653	0.7086	0.7636	0.076*	
C28	0.4413 (2)	0.8665 (2)	0.4126 (2)	0.0645 (8)	
H28A	0.4082	0.9322	0.3703	0.077*	
C12	0.3762 (2)	0.7579 (3)	0.7566 (2)	0.0748 (9)	
H12A	0.3615	0.7168	0.8204	0.090*	
H12B	0.3281	0.8266	0.7377	0.090*	
C10	0.2034 (2)	0.7150 (2)	0.7375 (2)	0.0624 (8)	
H10A	0.2032	0.7507	0.7769	0.075*	
C30	0.2349 (2)	0.9191 (2)	0.3947 (2)	0.0689 (9)	
H30A	0.2545	0.9438	0.3291	0.083*	
H30B	0.2465	0.9631	0.4245	0.083*	
C5	0.1268 (3)	0.5111 (3)	0.5683 (2)	0.0770 (10)	
H5A	0.1292	0.4689	0.5344	0.092*	
C26	0.5902 (2)	0.7451 (2)	0.4935 (2)	0.0593 (8)	
C32	0.0666 (2)	0.9902 (2)	0.3234 (2)	0.0676 (9)	
H32A	0.0927	1.0265	0.2671	0.081*	
C13	0.4781 (2)	0.7679 (3)	0.7527 (2)	0.0613 (8)	
C4	0.2055 (2)	0.5444 (2)	0.5673 (2)	0.0613 (8)	
H4A	0.2600	0.5261	0.5313	0.074*	
C22	0.5912 (2)	0.5769 (2)	0.6124 (2)	0.0659 (8)	
H22A	0.5593	0.5270	0.6463	0.079*	
C36	0.0884 (2)	0.8711 (2)	0.4832 (2)	0.0647 (8)	
H36A	0.1292	0.8273	0.5353	0.078*	
C7	0.0396 (2)	0.5987 (3)	0.6694 (2)	0.0761 (10)	
H7A	−0.0168	0.6181	0.7027	0.091*	
C33	−0.0319 (2)	1.0010 (2)	0.3292 (2)	0.0704 (9)	
H33A	−0.0726	1.0453	0.2772	0.084*	
C35	−0.0101 (2)	0.8811 (2)	0.4894 (2)	0.0693 (9)	
H35A	−0.0360	0.8439	0.5455	0.083*	
C16	0.6661 (2)	0.7860 (3)	0.7443 (3)	0.0688 (9)	
C14	0.5408 (3)	0.7126 (3)	0.8278 (2)	0.0744 (9)	
H14A	0.5197	0.6684	0.8829	0.089*	
C15	0.6343 (3)	0.7201 (3)	0.8244 (3)	0.0818 (10)	
H15A	0.6761	0.6806	0.8764	0.098*	
C27	0.5380 (2)	0.8428 (3)	0.4282 (2)	0.0710 (9)	
H27A	0.5711	0.8918	0.3954	0.085*	
C34	−0.0714 (2)	0.9465 (2)	0.4122 (2)	0.0650 (8)	
C18	0.5096 (3)	0.8351 (3)	0.6723 (2)	0.0743 (9)	
H18A	0.4671	0.8750	0.6209	0.089*	
C23	0.6873 (3)	0.5573 (3)	0.6319 (3)	0.0803 (10)	
H23A	0.7199	0.4944	0.6783	0.096*	
C25	0.6896 (2)	0.7216 (3)	0.5146 (3)	0.0766 (10)	
H25A	0.7238	0.7694	0.4811	0.092*	
C24	0.7376 (3)	0.6300 (3)	0.5835 (3)	0.0856 (11)	
H24A	0.8031	0.6165	0.5978	0.103*	
C6	0.0417 (3)	0.5386 (3)	0.6190 (3)	0.0867 (11)	
H6A	−0.0123	0.5161	0.6181	0.104*	
C19	0.7637 (3)	0.7936 (3)	0.7400 (3)	0.0975 (13)	
C17	0.6033 (3)	0.8437 (3)	0.6675 (3)	0.0784 (10)	
H17A	0.6244	0.8882	0.6126	0.094*	
C37	−0.1753 (3)	0.9587 (3)	0.4192 (3)	0.0902 (12)	
N1	−0.2582 (3)	0.9711 (3)	0.4230 (3)	0.1288 (14)	
N2	0.8419 (3)	0.7993 (3)	0.7366 (4)	0.1477 (17)	

### Atomic displacement parameters (Å^2^)

**Table:**

	*U*^11^	*U*^22^	*U*^33^	*U*^12^	*U*^13^	*U*^23^
O3	0.0591 (13)	0.0789 (15)	0.0610 (13)	−0.0161 (11)	0.0038 (10)	−0.0394 (12)
O4	0.0672 (14)	0.0583 (14)	0.0702 (14)	−0.0220 (11)	0.0007 (11)	−0.0152 (11)
C39	0.0581 (18)	0.0440 (16)	0.0450 (16)	−0.0112 (13)	−0.0009 (14)	−0.0131 (14)
C40	0.0576 (19)	0.0544 (18)	0.0520 (18)	−0.0158 (14)	−0.0053 (15)	−0.0160 (15)
C43	0.062 (2)	0.0501 (18)	0.0533 (19)	−0.0126 (15)	−0.0068 (16)	−0.0039 (15)
C48	0.066 (2)	0.0438 (17)	0.0470 (17)	−0.0146 (14)	−0.0018 (15)	−0.0054 (14)
C66	0.0531 (19)	0.064 (2)	0.0522 (18)	−0.0177 (16)	0.0011 (14)	−0.0239 (16)
C57	0.0615 (19)	0.060 (2)	0.0451 (17)	−0.0192 (15)	0.0059 (14)	−0.0254 (15)
C67	0.077 (2)	0.054 (2)	0.0571 (19)	−0.0201 (16)	0.0017 (16)	−0.0178 (16)
C58	0.066 (2)	0.082 (2)	0.0522 (19)	−0.0289 (18)	0.0149 (16)	−0.0383 (18)
C65	0.065 (2)	0.074 (2)	0.0549 (19)	−0.0164 (18)	0.0005 (16)	−0.0206 (17)
C68	0.071 (2)	0.0521 (19)	0.0490 (18)	−0.0206 (16)	0.0041 (16)	−0.0203 (16)
C41	0.068 (2)	0.065 (2)	0.062 (2)	−0.0130 (16)	−0.0043 (16)	−0.0293 (17)
C55	0.063 (2)	0.074 (2)	0.071 (2)	−0.0142 (17)	−0.0005 (18)	−0.0432 (19)
C71	0.067 (2)	0.064 (2)	0.059 (2)	−0.0223 (17)	0.0048 (17)	−0.0240 (18)
C63	0.059 (2)	0.101 (3)	0.061 (2)	−0.028 (2)	0.0092 (17)	−0.044 (2)
C44	0.067 (2)	0.063 (2)	0.072 (2)	−0.0175 (17)	−0.0169 (18)	0.0029 (18)
C38	0.074 (2)	0.060 (2)	0.062 (2)	−0.0173 (16)	−0.0022 (17)	−0.0253 (17)
C64	0.069 (2)	0.096 (3)	0.0494 (19)	−0.013 (2)	−0.0049 (16)	−0.028 (2)
C47	0.072 (2)	0.065 (2)	0.0538 (19)	−0.0091 (17)	−0.0032 (17)	−0.0151 (17)
C42	0.077 (2)	0.064 (2)	0.062 (2)	−0.0214 (17)	−0.0155 (18)	−0.0186 (17)
C51	0.078 (3)	0.095 (3)	0.065 (2)	−0.012 (2)	−0.0061 (19)	−0.037 (2)
C50	0.074 (2)	0.107 (3)	0.062 (2)	−0.027 (2)	0.0119 (19)	−0.043 (2)
C53	0.073 (2)	0.068 (2)	0.087 (3)	−0.0241 (19)	0.007 (2)	−0.010 (2)
C49	0.069 (2)	0.099 (3)	0.088 (3)	−0.0132 (19)	0.0066 (19)	−0.059 (2)
C52	0.061 (2)	0.060 (2)	0.086 (3)	−0.0101 (16)	−0.0047 (19)	−0.030 (2)
C74	0.081 (3)	0.084 (3)	0.069 (2)	−0.028 (2)	0.010 (2)	−0.0269 (19)
C69	0.070 (2)	0.080 (3)	0.070 (2)	−0.0226 (19)	0.0020 (18)	0.0069 (19)
C45	0.059 (2)	0.074 (2)	0.076 (3)	−0.0087 (19)	0.004 (2)	0.000 (2)
C73	0.101 (3)	0.076 (3)	0.080 (3)	−0.050 (2)	−0.023 (2)	0.003 (2)
C72	0.104 (3)	0.074 (3)	0.082 (3)	−0.043 (2)	−0.030 (2)	0.007 (2)
C46	0.074 (3)	0.080 (3)	0.064 (2)	−0.002 (2)	0.0036 (19)	−0.014 (2)
C54	0.079 (3)	0.068 (2)	0.066 (2)	−0.0145 (18)	−0.0033 (19)	−0.0138 (18)
N4	0.082 (2)	0.123 (3)	0.118 (3)	−0.044 (2)	0.006 (2)	−0.036 (2)
C59	0.091 (3)	0.094 (3)	0.089 (3)	−0.050 (2)	0.026 (2)	−0.035 (2)
C56	0.069 (3)	0.086 (3)	0.121 (3)	−0.020 (2)	−0.006 (2)	−0.040 (2)
C70	0.074 (2)	0.086 (3)	0.071 (2)	−0.031 (2)	0.009 (2)	0.005 (2)
C62	0.070 (3)	0.135 (4)	0.108 (3)	−0.040 (3)	0.011 (2)	−0.060 (3)
C60	0.112 (4)	0.127 (4)	0.128 (4)	−0.073 (3)	0.043 (3)	−0.043 (3)
N3	0.074 (2)	0.126 (3)	0.184 (4)	−0.022 (2)	−0.020 (3)	−0.043 (3)
C61	0.090 (4)	0.167 (6)	0.160 (5)	−0.072 (4)	0.034 (4)	−0.075 (4)
O2	0.0603 (13)	0.0508 (13)	0.0619 (13)	−0.0136 (10)	−0.0079 (10)	−0.0127 (10)
O1	0.0646 (13)	0.0876 (16)	0.0647 (14)	−0.0324 (11)	0.0146 (11)	−0.0454 (12)
C11	0.0532 (18)	0.0522 (18)	0.0495 (17)	−0.0158 (14)	0.0067 (14)	−0.0193 (14)
C20	0.0566 (18)	0.0490 (17)	0.0414 (15)	−0.0194 (14)	0.0091 (13)	−0.0210 (14)
C2	0.0528 (17)	0.0421 (16)	0.0400 (15)	−0.0121 (13)	0.0044 (13)	−0.0114 (13)
C8	0.0553 (19)	0.0473 (18)	0.0538 (18)	−0.0127 (14)	−0.0003 (15)	−0.0053 (15)
C21	0.0542 (18)	0.0563 (19)	0.0450 (16)	−0.0155 (14)	0.0034 (14)	−0.0257 (15)
C3	0.0515 (17)	0.0439 (16)	0.0476 (17)	−0.0166 (13)	0.0000 (13)	−0.0061 (14)
C1	0.0555 (18)	0.0493 (17)	0.0538 (18)	−0.0152 (14)	0.0042 (14)	−0.0175 (14)
C29	0.0531 (18)	0.0534 (19)	0.0475 (17)	−0.0159 (14)	−0.0036 (14)	−0.0170 (15)
C31	0.063 (2)	0.0485 (18)	0.0542 (19)	−0.0136 (14)	−0.0005 (15)	−0.0148 (15)
C9	0.060 (2)	0.060 (2)	0.061 (2)	−0.0140 (16)	0.0150 (16)	−0.0173 (16)
C28	0.073 (2)	0.059 (2)	0.0549 (19)	−0.0288 (17)	0.0006 (16)	−0.0067 (16)
C12	0.079 (2)	0.092 (3)	0.074 (2)	−0.0311 (19)	0.0112 (18)	−0.050 (2)
C10	0.064 (2)	0.062 (2)	0.061 (2)	−0.0156 (16)	0.0117 (16)	−0.0274 (17)
C30	0.069 (2)	0.055 (2)	0.070 (2)	−0.0132 (16)	0.0013 (17)	−0.0136 (17)
C5	0.086 (3)	0.078 (2)	0.079 (2)	−0.037 (2)	−0.001 (2)	−0.030 (2)
C26	0.058 (2)	0.065 (2)	0.0586 (19)	−0.0217 (16)	0.0026 (16)	−0.0253 (17)
C32	0.075 (2)	0.065 (2)	0.0475 (18)	−0.0105 (17)	−0.0009 (16)	−0.0122 (16)
C13	0.064 (2)	0.070 (2)	0.065 (2)	−0.0228 (17)	0.0056 (17)	−0.0400 (18)
C4	0.067 (2)	0.064 (2)	0.0585 (19)	−0.0261 (16)	0.0027 (16)	−0.0226 (16)
C22	0.061 (2)	0.064 (2)	0.065 (2)	−0.0114 (16)	−0.0002 (16)	−0.0211 (18)
C36	0.069 (2)	0.053 (2)	0.061 (2)	−0.0094 (16)	−0.0079 (17)	−0.0138 (16)
C7	0.056 (2)	0.076 (2)	0.084 (3)	−0.0247 (18)	0.0075 (18)	−0.014 (2)
C33	0.069 (2)	0.069 (2)	0.058 (2)	−0.0077 (17)	−0.0107 (17)	−0.0143 (18)
C35	0.076 (2)	0.055 (2)	0.065 (2)	−0.0172 (17)	0.0019 (18)	−0.0108 (17)
C16	0.064 (2)	0.067 (2)	0.085 (3)	−0.0210 (18)	0.007 (2)	−0.039 (2)
C14	0.071 (2)	0.079 (2)	0.071 (2)	−0.0245 (19)	0.0036 (19)	−0.0241 (19)
C15	0.073 (2)	0.084 (3)	0.079 (3)	−0.016 (2)	−0.011 (2)	−0.022 (2)
C27	0.071 (2)	0.076 (2)	0.067 (2)	−0.0402 (19)	0.0097 (18)	−0.0160 (19)
C34	0.064 (2)	0.054 (2)	0.073 (2)	−0.0085 (16)	−0.0065 (18)	−0.0247 (18)
C18	0.080 (2)	0.084 (3)	0.062 (2)	−0.029 (2)	−0.0035 (18)	−0.0247 (19)
C23	0.065 (2)	0.080 (3)	0.088 (3)	−0.009 (2)	−0.006 (2)	−0.030 (2)
C25	0.059 (2)	0.090 (3)	0.089 (3)	−0.0289 (19)	0.0112 (19)	−0.039 (2)
C24	0.055 (2)	0.101 (3)	0.108 (3)	−0.011 (2)	−0.005 (2)	−0.054 (3)
C6	0.067 (2)	0.087 (3)	0.112 (3)	−0.034 (2)	−0.003 (2)	−0.034 (2)
C19	0.075 (3)	0.087 (3)	0.141 (4)	−0.026 (2)	0.005 (3)	−0.054 (3)
C17	0.101 (3)	0.072 (2)	0.072 (2)	−0.045 (2)	0.016 (2)	−0.025 (2)
C37	0.074 (3)	0.076 (3)	0.117 (3)	−0.019 (2)	−0.009 (2)	−0.030 (2)
N1	0.082 (2)	0.106 (3)	0.177 (4)	−0.021 (2)	−0.014 (3)	−0.030 (3)
N2	0.083 (3)	0.128 (3)	0.244 (5)	−0.043 (2)	0.005 (3)	−0.073 (3)

### Geometric parameters (Å, °)

**Table:**

O3—C40	1.373 (3)	O2—C29	1.386 (3)
O3—C49	1.419 (3)	O2—C30	1.425 (3)
O4—C66	1.375 (3)	O1—C11	1.372 (3)
O4—C67	1.416 (3)	O1—C12	1.405 (3)
C39—C40	1.374 (4)	C11—C2	1.370 (3)
C39—C48	1.429 (4)	C11—C10	1.402 (4)
C39—C38	1.513 (4)	C20—C29	1.376 (4)
C40—C41	1.404 (4)	C20—C21	1.426 (4)
C43—C44	1.417 (4)	C20—C1	1.516 (3)
C43—C42	1.409 (4)	C2—C3	1.426 (3)
C43—C48	1.425 (4)	C2—C1	1.532 (3)
C48—C47	1.411 (4)	C8—C9	1.413 (4)
C66—C57	1.374 (4)	C8—C7	1.406 (4)
C66—C65	1.403 (4)	C8—C3	1.425 (4)
C57—C58	1.423 (4)	C21—C22	1.407 (4)
C57—C38	1.512 (4)	C21—C26	1.431 (4)
C67—C68	1.494 (4)	C3—C4	1.411 (4)
C67—H67A	0.9700	C1—H1A	0.9700
C67—H67B	0.9700	C1—H1B	0.9700
C58—C63	1.427 (4)	C29—C28	1.396 (4)
C58—C59	1.411 (4)	C31—C36	1.383 (4)
C65—C64	1.358 (4)	C31—C32	1.380 (4)
C65—H65A	0.9300	C31—C30	1.500 (4)
C68—C73	1.355 (4)	C9—C10	1.365 (4)
C68—C69	1.366 (4)	C9—H9A	0.9300
C41—C42	1.360 (4)	C28—C27	1.342 (4)
C41—H41A	0.9300	C28—H28A	0.9300
C55—C54	1.360 (4)	C12—C13	1.502 (4)
C55—C50	1.392 (4)	C12—H12A	0.9700
C55—C49	1.500 (4)	C12—H12B	0.9700
C71—C70	1.370 (4)	C10—H10A	0.9300
C71—C72	1.364 (4)	C30—H30A	0.9700
C71—C74	1.444 (5)	C30—H30B	0.9700
C63—C64	1.396 (4)	C5—C4	1.352 (4)
C63—C62	1.417 (5)	C5—C6	1.401 (5)
C44—C45	1.354 (5)	C5—H5A	0.9300
C44—H44A	0.9300	C26—C25	1.401 (4)
C38—H38A	0.9700	C26—C27	1.408 (4)
C38—H38B	0.9700	C32—C33	1.364 (4)
C64—H64A	0.9300	C32—H32A	0.9300
C47—C46	1.366 (4)	C13—C18	1.378 (4)
C47—H47A	0.9300	C13—C14	1.359 (4)
C42—H42A	0.9300	C4—H4A	0.9300
C51—C50	1.370 (4)	C22—C23	1.359 (4)
C51—C52	1.395 (4)	C22—H22A	0.9300
C51—H51A	0.9300	C36—C35	1.368 (4)
C50—H50A	0.9300	C36—H36A	0.9300
C53—C54	1.368 (4)	C7—C6	1.354 (5)
C53—C52	1.377 (4)	C7—H7A	0.9300
C53—H53A	0.9300	C33—C34	1.383 (4)
C49—H49A	0.9700	C33—H33A	0.9300
C49—H49B	0.9700	C35—C34	1.386 (4)
C52—C56	1.425 (5)	C35—H35A	0.9300
C74—N4	1.144 (4)	C16—C15	1.369 (4)
C69—C70	1.375 (4)	C16—C17	1.381 (5)
C69—H69A	0.9300	C16—C19	1.427 (5)
C45—C46	1.382 (5)	C14—C15	1.369 (4)
C45—H45A	0.9300	C14—H14A	0.9300
C73—C72	1.373 (4)	C15—H15A	0.9300
C73—H73A	0.9300	C27—H27A	0.9300
C72—H72A	0.9300	C34—C37	1.437 (5)
C46—H46A	0.9300	C18—C17	1.376 (4)
C54—H54A	0.9300	C18—H18A	0.9300
C59—C60	1.356 (5)	C23—C24	1.390 (5)
C59—H59A	0.9300	C23—H23A	0.9300
C56—N3	1.139 (4)	C25—C24	1.366 (5)
C70—H70A	0.9300	C25—H25A	0.9300
C62—C61	1.348 (6)	C24—H24A	0.9300
C62—H62A	0.9300	C6—H6A	0.9300
C60—C61	1.382 (6)	C19—N2	1.141 (4)
C60—H60A	0.9300	C17—H17A	0.9300
C61—H61A	0.9300	C37—N1	1.141 (4)
			
C40—O3—C49	118.1 (2)	C29—O2—C30	118.0 (2)
C66—O4—C67	119.2 (2)	C11—O1—C12	119.2 (2)
C40—C39—C48	118.8 (3)	C2—C11—O1	116.2 (2)
C40—C39—C38	121.4 (3)	C2—C11—C10	121.8 (3)
C48—C39—C38	119.9 (3)	O1—C11—C10	122.0 (3)
O3—C40—C39	116.4 (2)	C29—C20—C21	118.6 (2)
O3—C40—C41	121.9 (3)	C29—C20—C1	119.8 (2)
C39—C40—C41	121.7 (3)	C21—C20—C1	121.4 (2)
C44—C43—C42	121.9 (3)	C11—C2—C3	118.4 (2)
C44—C43—C48	119.5 (3)	C11—C2—C1	121.5 (2)
C42—C43—C48	118.5 (3)	C3—C2—C1	119.9 (2)
C39—C48—C43	119.5 (3)	C9—C8—C7	121.5 (3)
C39—C48—C47	123.9 (3)	C9—C8—C3	118.4 (3)
C43—C48—C47	116.6 (3)	C7—C8—C3	120.1 (3)
C57—C66—O4	116.3 (3)	C22—C21—C20	124.0 (3)
C57—C66—C65	121.8 (3)	C22—C21—C26	116.7 (3)
O4—C66—C65	121.8 (3)	C20—C21—C26	119.2 (3)
C66—C57—C58	118.8 (3)	C4—C3—C8	116.6 (3)
C66—C57—C38	119.5 (3)	C4—C3—C2	123.4 (3)
C58—C57—C38	121.6 (3)	C8—C3—C2	120.0 (3)
O4—C67—C68	110.4 (2)	C20—C1—C2	118.0 (2)
O4—C67—H67A	109.6	C20—C1—H1A	107.8
C68—C67—H67A	109.6	C2—C1—H1A	107.8
O4—C67—H67B	109.6	C20—C1—H1B	107.8
C68—C67—H67B	109.6	C2—C1—H1B	107.8
H67A—C67—H67B	108.1	H1A—C1—H1B	107.1
C63—C58—C57	119.1 (3)	C20—C29—O2	116.0 (2)
C63—C58—C59	117.2 (3)	C20—C29—C28	121.6 (3)
C57—C58—C59	123.7 (3)	O2—C29—C28	122.4 (3)
C64—C65—C66	119.7 (3)	C36—C31—C32	119.2 (3)
C64—C65—H65A	120.1	C36—C31—C30	120.5 (3)
C66—C65—H65A	120.1	C32—C31—C30	120.2 (3)
C73—C68—C69	118.0 (3)	C10—C9—C8	120.9 (3)
C73—C68—C67	122.5 (3)	C10—C9—H9A	119.5
C69—C68—C67	119.5 (3)	C8—C9—H9A	119.5
C42—C41—C40	119.8 (3)	C27—C28—C29	120.7 (3)
C42—C41—H41A	120.1	C27—C28—H28A	119.7
C40—C41—H41A	120.1	C29—C28—H28A	119.7
C54—C55—C50	119.5 (3)	O1—C12—C13	107.4 (2)
C54—C55—C49	120.8 (3)	O1—C12—H12A	110.2
C50—C55—C49	119.6 (3)	C13—C12—H12A	110.2
C70—C71—C72	118.4 (3)	O1—C12—H12B	110.2
C70—C71—C74	119.7 (3)	C13—C12—H12B	110.2
C72—C71—C74	121.9 (3)	H12A—C12—H12B	108.5
C64—C63—C58	119.2 (3)	C9—C10—C11	120.1 (3)
C64—C63—C62	121.5 (4)	C9—C10—H10A	120.0
C58—C63—C62	119.3 (4)	C11—C10—H10A	120.0
C45—C44—C43	120.9 (3)	O2—C30—C31	109.4 (2)
C45—C44—H44A	119.6	O2—C30—H30A	109.8
C43—C44—H44A	119.6	C31—C30—H30A	109.8
C57—C38—C39	119.8 (2)	O2—C30—H30B	109.8
C57—C38—H38A	107.4	C31—C30—H30B	109.8
C39—C38—H38A	107.4	H30A—C30—H30B	108.2
C57—C38—H38B	107.4	C4—C5—C6	121.8 (3)
C39—C38—H38B	107.4	C4—C5—H5A	119.1
H38A—C38—H38B	106.9	C6—C5—H5A	119.1
C65—C64—C63	121.3 (3)	C25—C26—C27	121.8 (3)
C65—C64—H64A	119.4	C25—C26—C21	119.5 (3)
C63—C64—H64A	119.4	C27—C26—C21	118.6 (3)
C46—C47—C48	122.3 (3)	C33—C32—C31	120.7 (3)
C46—C47—H47A	118.9	C33—C32—H32A	119.7
C48—C47—H47A	118.9	C31—C32—H32A	119.7
C41—C42—C43	121.5 (3)	C18—C13—C14	118.5 (3)
C41—C42—H42A	119.3	C18—C13—C12	120.2 (3)
C43—C42—H42A	119.3	C14—C13—C12	121.3 (3)
C50—C51—C52	120.2 (3)	C5—C4—C3	121.5 (3)
C50—C51—H51A	119.9	C5—C4—H4A	119.2
C52—C51—H51A	119.9	C3—C4—H4A	119.2
C51—C50—C55	119.8 (3)	C23—C22—C21	122.2 (3)
C51—C50—H50A	120.1	C23—C22—H22A	118.9
C55—C50—H50A	120.1	C21—C22—H22A	118.9
C54—C53—C52	120.0 (3)	C31—C36—C35	120.4 (3)
C54—C53—H53A	120.0	C31—C36—H36A	119.8
C52—C53—H53A	120.0	C35—C36—H36A	119.8
O3—C49—C55	108.1 (2)	C6—C7—C8	121.4 (3)
O3—C49—H49A	110.1	C6—C7—H7A	119.3
C55—C49—H49A	110.1	C8—C7—H7A	119.3
O3—C49—H49B	110.1	C32—C33—C34	120.2 (3)
C55—C49—H49B	110.1	C32—C33—H33A	119.9
H49A—C49—H49B	108.4	C34—C33—H33A	119.9
C53—C52—C51	119.2 (3)	C36—C35—C34	120.1 (3)
C53—C52—C56	119.9 (4)	C36—C35—H35A	119.9
C51—C52—C56	120.8 (3)	C34—C35—H35A	119.9
N4—C74—C71	179.2 (4)	C15—C16—C17	119.3 (3)
C68—C69—C70	121.0 (3)	C15—C16—C19	120.2 (4)
C68—C69—H69A	119.5	C17—C16—C19	120.5 (4)
C70—C69—H69A	119.5	C15—C14—C13	121.7 (3)
C44—C45—C46	120.5 (3)	C15—C14—H14A	119.1
C44—C45—H45A	119.8	C13—C14—H14A	119.1
C46—C45—H45A	119.8	C14—C15—C16	119.9 (3)
C68—C73—C72	121.7 (3)	C14—C15—H15A	120.0
C68—C73—H73A	119.1	C16—C15—H15A	120.0
C72—C73—H73A	119.1	C28—C27—C26	121.3 (3)
C71—C72—C73	120.3 (3)	C28—C27—H27A	119.4
C71—C72—H72A	119.8	C26—C27—H27A	119.4
C73—C72—H72A	119.8	C33—C34—C35	119.4 (3)
C47—C46—C45	120.2 (3)	C33—C34—C37	120.4 (3)
C47—C46—H46A	119.9	C35—C34—C37	120.2 (3)
C45—C46—H46A	119.9	C13—C18—C17	120.6 (3)
C55—C54—C53	121.3 (3)	C13—C18—H18A	119.7
C55—C54—H54A	119.4	C17—C18—H18A	119.7
C53—C54—H54A	119.4	C22—C23—C24	120.8 (3)
C60—C59—C58	121.2 (4)	C22—C23—H23A	119.6
C60—C59—H59A	119.4	C24—C23—H23A	119.6
C58—C59—H59A	119.4	C24—C25—C26	121.5 (3)
N3—C56—C52	179.3 (5)	C24—C25—H25A	119.3
C71—C70—C69	120.5 (3)	C26—C25—H25A	119.3
C71—C70—H70A	119.7	C25—C24—C23	119.3 (3)
C69—C70—H70A	119.7	C25—C24—H24A	120.4
C61—C62—C63	120.8 (4)	C23—C24—H24A	120.4
C61—C62—H62A	119.6	C7—C6—C5	118.6 (3)
C63—C62—H62A	119.6	C7—C6—H6A	120.7
C59—C60—C61	121.4 (5)	C5—C6—H6A	120.7
C59—C60—H60A	119.3	N2—C19—C16	179.8 (5)
C61—C60—H60A	119.3	C18—C17—C16	119.9 (3)
C62—C61—C60	120.1 (5)	C18—C17—H17A	120.0
C62—C61—H61A	119.9	C16—C17—H17A	120.0
C60—C61—H61A	119.9	N1—C37—C34	178.2 (5)
			
C49—O3—C40—C39	−174.5 (3)	C12—O1—C11—C2	179.6 (3)
C49—O3—C40—C41	8.4 (4)	C12—O1—C11—C10	−0.7 (4)
C48—C39—C40—O3	−174.9 (2)	O1—C11—C2—C3	172.8 (2)
C38—C39—C40—O3	4.0 (4)	C10—C11—C2—C3	−6.9 (4)
C48—C39—C40—C41	2.2 (4)	O1—C11—C2—C1	−3.2 (4)
C38—C39—C40—C41	−178.9 (3)	C10—C11—C2—C1	177.1 (2)
C40—C39—C48—C43	−4.2 (4)	C29—C20—C21—C22	173.6 (3)
C38—C39—C48—C43	176.9 (2)	C1—C20—C21—C22	−10.7 (4)
C40—C39—C48—C47	176.2 (3)	C29—C20—C21—C26	−3.1 (4)
C38—C39—C48—C47	−2.8 (4)	C1—C20—C21—C26	172.7 (2)
C44—C43—C48—C39	−176.2 (3)	C9—C8—C3—C4	176.4 (2)
C42—C43—C48—C39	2.9 (4)	C7—C8—C3—C4	−1.1 (4)
C44—C43—C48—C47	3.5 (4)	C9—C8—C3—C2	−2.0 (4)
C42—C43—C48—C47	−177.4 (3)	C7—C8—C3—C2	−179.5 (3)
C67—O4—C66—C57	−179.7 (2)	C11—C2—C3—C4	−171.9 (3)
C67—O4—C66—C65	2.7 (4)	C1—C2—C3—C4	4.2 (4)
O4—C66—C57—C58	−177.0 (2)	C11—C2—C3—C8	6.3 (4)
C65—C66—C57—C58	0.6 (4)	C1—C2—C3—C8	−177.6 (2)
O4—C66—C57—C38	−1.2 (4)	C29—C20—C1—C2	−62.2 (3)
C65—C66—C57—C38	176.5 (3)	C21—C20—C1—C2	122.1 (3)
C66—O4—C67—C68	177.6 (2)	C11—C2—C1—C20	−39.5 (4)
C66—C57—C58—C63	2.5 (4)	C3—C2—C1—C20	144.5 (2)
C38—C57—C58—C63	−173.2 (2)	C21—C20—C29—O2	179.1 (2)
C66—C57—C58—C59	−178.5 (3)	C1—C20—C29—O2	3.3 (4)
C38—C57—C58—C59	5.7 (4)	C21—C20—C29—C28	2.3 (4)
C57—C66—C65—C64	−1.4 (4)	C1—C20—C29—C28	−173.6 (2)
O4—C66—C65—C64	176.1 (3)	C30—O2—C29—C20	165.7 (2)
O4—C67—C68—C73	−22.2 (4)	C30—O2—C29—C28	−17.5 (4)
O4—C67—C68—C69	160.6 (3)	C7—C8—C9—C10	175.5 (3)
O3—C40—C41—C42	178.0 (3)	C3—C8—C9—C10	−2.0 (4)
C39—C40—C41—C42	1.0 (4)	C20—C29—C28—C27	0.1 (4)
C57—C58—C63—C64	−4.9 (4)	O2—C29—C28—C27	−176.6 (3)
C59—C58—C63—C64	176.0 (3)	C11—O1—C12—C13	179.3 (2)
C57—C58—C63—C62	176.8 (3)	C8—C9—C10—C11	1.6 (4)
C59—C58—C63—C62	−2.3 (4)	C2—C11—C10—C9	3.0 (4)
C42—C43—C44—C45	179.3 (3)	O1—C11—C10—C9	−176.6 (3)
C48—C43—C44—C45	−1.6 (4)	C29—O2—C30—C31	−168.4 (2)
C66—C57—C38—C39	58.2 (4)	C36—C31—C30—O2	42.6 (4)
C58—C57—C38—C39	−126.0 (3)	C32—C31—C30—O2	−140.2 (3)
C40—C39—C38—C57	41.0 (4)	C22—C21—C26—C25	1.6 (4)
C48—C39—C38—C57	−140.1 (3)	C20—C21—C26—C25	178.5 (3)
C66—C65—C64—C63	−1.1 (5)	C22—C21—C26—C27	−175.2 (3)
C58—C63—C64—C65	4.3 (5)	C20—C21—C26—C27	1.7 (4)
C62—C63—C64—C65	−177.5 (3)	C36—C31—C32—C33	0.7 (5)
C39—C48—C47—C46	176.9 (3)	C30—C31—C32—C33	−176.5 (3)
C43—C48—C47—C46	−2.8 (4)	O1—C12—C13—C18	69.1 (4)
C40—C41—C42—C43	−2.4 (5)	O1—C12—C13—C14	−111.7 (3)
C44—C43—C42—C41	179.4 (3)	C6—C5—C4—C3	1.8 (5)
C48—C43—C42—C41	0.4 (4)	C8—C3—C4—C5	−0.7 (4)
C52—C51—C50—C55	0.5 (5)	C2—C3—C4—C5	177.6 (3)
C54—C55—C50—C51	−1.0 (5)	C20—C21—C22—C23	−177.5 (3)
C49—C55—C50—C51	−177.7 (3)	C26—C21—C22—C23	−0.9 (4)
C40—O3—C49—C55	178.5 (3)	C32—C31—C36—C35	−0.3 (5)
C54—C55—C49—O3	119.6 (3)	C30—C31—C36—C35	176.9 (3)
C50—C55—C49—O3	−63.8 (4)	C9—C8—C7—C6	−175.6 (3)
C54—C53—C52—C51	−2.1 (5)	C3—C8—C7—C6	1.9 (5)
C54—C53—C52—C56	−179.2 (3)	C31—C32—C33—C34	−0.7 (5)
C50—C51—C52—C53	1.0 (5)	C31—C36—C35—C34	−0.2 (5)
C50—C51—C52—C56	178.1 (3)	C18—C13—C14—C15	−1.7 (5)
C73—C68—C69—C70	0.5 (5)	C12—C13—C14—C15	179.2 (3)
C67—C68—C69—C70	177.7 (3)	C13—C14—C15—C16	1.0 (5)
C43—C44—C45—C46	−1.3 (5)	C17—C16—C15—C14	−0.4 (5)
C69—C68—C73—C72	−1.7 (5)	C19—C16—C15—C14	−179.4 (3)
C67—C68—C73—C72	−178.9 (3)	C29—C28—C27—C26	−1.5 (5)
C70—C71—C72—C73	1.3 (5)	C25—C26—C27—C28	−176.1 (3)
C74—C71—C72—C73	179.2 (3)	C21—C26—C27—C28	0.7 (5)
C68—C73—C72—C71	0.8 (6)	C32—C33—C34—C35	0.2 (5)
C48—C47—C46—C45	0.0 (5)	C32—C33—C34—C37	179.1 (3)
C44—C45—C46—C47	2.1 (5)	C36—C35—C34—C33	0.2 (5)
C50—C55—C54—C53	−0.1 (5)	C36—C35—C34—C37	−178.7 (3)
C49—C55—C54—C53	176.5 (3)	C14—C13—C18—C17	1.8 (5)
C52—C53—C54—C55	1.7 (5)	C12—C13—C18—C17	−179.1 (3)
C63—C58—C59—C60	2.9 (5)	C21—C22—C23—C24	0.5 (5)
C57—C58—C59—C60	−176.1 (3)	C27—C26—C25—C24	174.6 (3)
C72—C71—C70—C69	−2.5 (5)	C21—C26—C25—C24	−2.1 (5)
C74—C71—C70—C69	179.6 (3)	C26—C25—C24—C23	1.7 (5)
C68—C69—C70—C71	1.7 (6)	C22—C23—C24—C25	−0.9 (5)
C64—C63—C62—C61	−177.9 (4)	C8—C7—C6—C5	−0.8 (5)
C58—C63—C62—C61	0.3 (6)	C4—C5—C6—C7	−1.1 (5)
C58—C59—C60—C61	−1.5 (7)	C13—C18—C17—C16	−1.2 (5)
C63—C62—C61—C60	1.1 (7)	C15—C16—C17—C18	0.5 (5)
C59—C60—C61—C62	−0.5 (8)	C19—C16—C17—C18	179.5 (3)

### Hydrogen-bond geometry (Å, °)

**Table:**

*D*—H···*A*	*D*—H	H···*A*	*D*···*A*	*D*—H···*A*
O73—H73A···O4	0.93	2.44	2.763 (5)	100
C22—H22A···Cg1^i^	0.93	2.88	3.755 (3)	158
C36—H36A···Cg2	0.93	2.57	3.454 (3)	159
C73—H73A···Cg1	0.93	2.78	3.698 (4)	168

Symmetry codes: (i) −*x*+1, −*y*+1, −*z*+1.
